# 

**Published:** 1887-04-30

**Authors:** 


					H\
PRICE ONE PENNY. hc-zL
?0A 31. tfnl. TT X
7 April^Oth, 1887./
. * ' *,M> General JPost Office
?? a SewspapeiyJ
f
Per Annum, 6s. 6d.,post free.
Monthly Parts, 6d.; post free, 7id.
Ditto per Ann., 7s. 6d., post free.
?*** ^s^rifoziasi: tfospi ^ ^B3Sa&a!^o^g /^anA
8n Institution, jfamtlg, aitft^k, $aroc1)iaI journal of
j^osyitals. &gt)tutng, & alt agencies for tfte (Hare of tfie g>icfe. ffritictem & ?etog. ^1^1X81

				

